# New Appliances and Things Medical

**Published:** 1896-05-02

**Authors:** 


### NEW APPLIANCES AND THINGS MEDICAL.

### VINOLIA.

(Blondeau and Co., Malden Crescent, N.W.)

If people were asked the question, "What is Vinolia?" many would be the answers given. Some remembering how pleasant it had seemed when applied to rough skins and hot places, and how readily it washed off when used to puff the baby, leaving no lumps, no grittiness, and no irritation, would say it was an extremely good puff "powder"; others would say it was a "cream," useful whether the skin was rough, soothing when in cold weather the hands are chapped, and emollient when in the blaze of a July sun the face is burned, the cheeks are skinned, and the nose is peeling; others again would say, simply and shortly, without any more palaver, "It is a soap." Even these would have to confess that, according to circumstances, it is a "toilet soap," or a "shaving stick," or a "shaving foam," or a "medicated soap," but that whatever form it takes the soap is good, and, while it tends to lather, does not dissolve the skin. Vinolia then seems to be a very different thing according to the way one looks at it, and, in fact, the truth is that Vinolia is but a name, a sort of registered trade mark, which cannot be imitated by anyone under heavy penalties, and assures everyone who sees it on any article that that article is made by Messrs. Blondeau, and is guaranteed by them to be of good quality. And, indeed, what higher recommendation could one ask for? Vinolia powder is said to consist largely of boracic acid, the antiseptic qualities of which are sufficiently well known, and those who are acquainted with the silky softness of the material even when crystallised upon lint will understand how, when it has been by infinite patience brought to the form of a literally impalpable powder, it serves as a most efficacious and agreeable "puff." In an article of this kind purity of chemical composition is but one item in its nature. The physical condition in which it exists is of equal importance, and it has been found in the preparation of Vinolia powder that by subjecting it to laborious trituration a product has been obtained, the virtues of which, as a dusting powder, are far greater than could be expressed by its mere chemical formula. Vinolia soap may be of many kinds. It may be cheap—three tablets for tenpence—and yet smooth, and soft, and pleasant to the skin, and for all its cheapness scented with good stuff; or it may cost six and threepence for three tablets, which, after all, is a good deal under half-a-crown a piece, and for that one gets what is really the purest soap one can desire—a "Vestal Soap," as it is called—the very name suggesting purity, a soap made of pure fat, pure alkali, and pure scent. On the good basis also presented by Vinolia soap are built up various kinds of medicated soaps—coal tar Vinolia soap, terebene Vinolia soap, carbolic Vinolia soap, sulphur Vinolia soap—in all of which we have the satisfaction of feeling that the medicament is added to an innocuous basis, instead of being made by its odour to cover the disagreeables of an inferior soap; and surely it is important in applying medicine to the skin to be sure that the basis with which it is mixed is pure. What, then, are the peculiarities of Vinolia soap of whatever grade? The answer is pure fat, pure scent, and pure alkali, and as to the latter it must be added—not too much of it. Another virtue in Vinolia is one which one would like to meet with more often in this world, namely, its honesty. In making soap the problem too often is to find out how much water it will carry, and for this purpose many makers add substances which, whatever else they be, certainly are not soap. From such adulterations Vinolia is always free, and anyone who has seen the trouble taken at Messrs. Blondeau's works to get rid of every particle of water, except just what is necessary to make the soap cohesive, will agree that purchasers of Vinolia soap get for their money soap, and nothing else—except, that is, the pretty boxes in which it is packed up.

